# Double Interphalangeal Joint Dislocation of the Finger in Adolescent Athletes: A Review of Two Cases and the Relevant Literature

**DOI:** 10.1155/2021/6684553

**Published:** 2021-03-15

**Authors:** Angelo V. Vasiliadis, Alexandros Maris, Maria Tsatlidou, Edward D. R. Bray, Dimitrios Metaxiotis

**Affiliations:** ^1^2nd Orthopaedic Department, General Hospital of Thessaloniki “Papageorgiou”, Ring Road-N. Eukarpia, 56403 Thessaloniki, Greece; ^2^School of Medicine, Aristotle University of Thessaloniki, University Campus, 54124 Thessaloniki, Greece; ^3^Department of Trauma and Orthopaedics, St George's University Hospital, London, UK; ^4^Department of Orthopaedics, Great Ormond Street Hospital, London, UK

## Abstract

Double interphalangeal joint dislocation of the same finger is a rare condition. We report two cases of adolescent athletes with distal and proximal interphalangeal joint dislocation. The diagnosis was confirmed with plain radiograph, while anatomical reduction was easily obtained with gentle longitudinal traction. A simple immobilization of the injured finger was applied by buddy taping for two weeks. Early mobilization as tolerated was recommended, and they have made a full return to their previous status of activities within 5 months. We also provide a review of the literature detailing demographic characteristics, cause and mechanism of injury with associated injuries, treatment options, and functional outcomes in this population.

## 1. Introduction

Single interphalangeal joint dislocations are common upper extremity dislocations. However, double dislocation of the distal interphalangeal (DIP) and proximal interphalangeal (PIP) joints of the fingers seems to be rare [1]. This type of injury was first reported by Bartels in 1874 [2]. Since then, only case reports have been reported in the literature [3–6]. In this paper, two cases of double dislocation of both DIP and PIP joints in a single finger are reported, alongside with a panorama of the current literature.

## 2. Case Report

### 2.1. Case 1

A 13-year-old male presented in the emergency department with his little finger of the nondominant hand injured while playing basketball. An axial compression with an excessive hyperextension was applied to the tip of his finger while he tried to steal the ball during the game.

**Figure 1:**
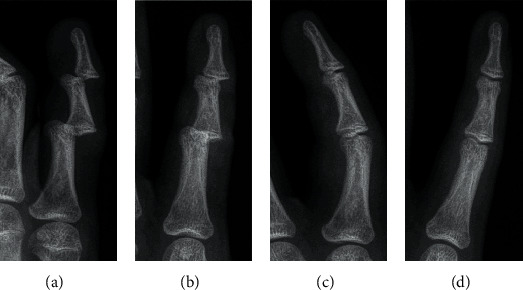
Anteroposterior and lateral radiographs (a, b) of the index finger showing dorsal dislocation of both the proximal and distal interphalangeal joints, without any bony avulsion fracture. Postreduction anteroposterior and lateral radiographs (c, d) confirm that all joints are well reduced.

On physical examination, the finger was found to be deformed, swollen and very painful in performing any movement. There were no signs of neurovascular damage or skin lesion. Initial plain radiographs demonstrated a dorsal dislocation of PIP and DIP joints of his little finger in a step-ladder deformity (Figure 1). Anatomical reduction was easily obtained with a simple and gentle longitudinal traction. Following reduction, both joints were stable and confirmed on plain radiographs with no apparent any bony avulsion fragment or fracture (Figure 1). After that, buddy taping with the ring finger was applied for two weeks, and the patient was advised to mobilize the PIP and DIP joints as long as the discomfort allowed him.

At 3 weeks of follow-up, the buddy taping was removed, and he had full extension of both joints, with flexion of the DIP joint to 50° and the PIP joint to 90°. At 6 weeks of follow-up, the patient had full active range of motion. Within 3 months after the reduction, the patient returned to previous level of musical activities (piano playing) without limitation of motion.

### 2.2. Case 2

A 16-year-old male sustained a hyperextension injury in his index finger of the dominant had while playing football (goalkeeper). The ball had hit to the tip of his finger while he tried to get the ball away from his goal post.

**Figure 2:**
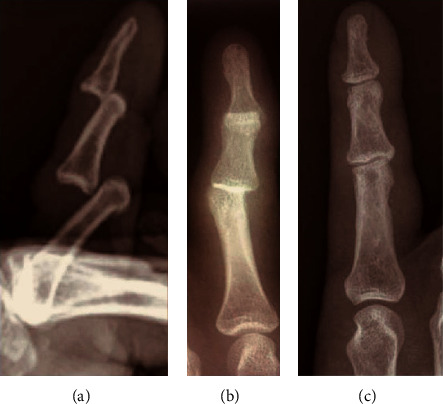
Anteroposterior and lateral radiographs (a, b) demonstrating dorsal dislocation of both proximal and distal interphalangeal joints, without any bony avulsion fracture. Postreduction radiograph (c) confirms that all joints are well reduced.

On physical examination, the finger was markedly deformed with swelling and difficulty on moving the finger. Neurovascular examination was normal. Initial plain radiographs demonstrated a dorsal dislocation of both the distal and proximal interphalangeal joints (Figure 2). Without the use of local anesthesia, reduction of both the dislocations was easily achieved, by longitudinal traction and pressure on the dorsal aspect of the distal phalanx. Plain radiographs confirmed successful reduction without any bony avulsion fracture (Figure 2). Both joints were stable, and the index finger was immobilized with the middle finger for two weeks.

At 3 weeks of follow-up, the flexion of the DIP and PIP joints was 45° and 75°, respectively, while extension was normal in both joints. At 6 weeks of follow-up, the patient had almost full painless active range of motion. 5 months after reduction, he returned to previous level of sports activities.

## 3. Discussion

Both dislocations of both PIP and DIP joints of a finger is a rare condition. Dislocations of proximal interphalangeal joint of the finger are relatively uncommon due to the protected position of this joint in the hand [2]. While in most of the cases dislocations appear in males, aged near the third decade of their life, our case report highlights two cases of both dislocations of PIP and DIP joints among adolescent athletes (below 16 years of age). The uniqueness is that these are among the youngest cases in the international literature. Sports activities, such as basketball, football, and softball, are by far the commonest cause of injury. Falls is the second common reason implicated in some of the cases, followed by work-related injuries (Table 1) [7–10]. A classic “step-ladder” deformity of the affected finger will be obvious, and the diagnosis confirmed with a plain radiograph [3]. The little finger of the dominant hand is the most common digit involved, followed by the ring finger (Table 1). The increased mobility, the lack of protection from the surrounding fingers, and the weakness of the articular capsule and collateral ligaments of the interphalangeal joints may be posed as logical explanations [2].

All the information gained about the mechanism of this injury is related to be a traumatic hyperextension [10, 11]. Firstly, an axial compression force to the tip of the finger results in an excessive hyperextension at the DIP joint, causing injury of the volar articular capsule and allowing dorsal dislocation of the DIP joint. Subsequently, this force was transmitted to the middle phalanx, which resulted in similar injury of the volar articular capsule of the PIP joint causing a second dorsal dislocation at the proximal joint [3, 8]. Thus, it is obvious that it is not a “simultaneous” dislocation, but two separate actions that occur in a quick succession and result to this double dislocation of both distal and proximal joints. Also, this type of injury may be accompanied with avulsion fracture of the volar side of the base middle phalanx [10, 12] and rupture of the central slip of the extensor tendon [5]. In our cases, there were not any accompanying avulsion fractures or tendon injuries.

In most dislocations, closed reduction is the treatment of choice if there is not interposition of periarticular soft tissues that prohibits this action and can be easily achieved by longitudinal traction and pressure on the dislocated base of the phalanx, with [2, 6, 13] or without anesthesia [4, 8]. However, patients with neglected injuries, open dislocations, ligamentous injuries, and associated fractures or flexor tendon injuries were not so fortunate, especially in cases where larger defects involving more than 50% of the articular surface may have worse outcomes after conservative treatment and may be better treated with fragment surgical fixation in order to stabilize the joint and avoid limitations of the range of motion [9, 10]. Static stabilizers, such as collateral ligaments, are the main stabilizers of the DIP and PIP joints. Its attachment in the middle phalanx is crucial to the stability of the PIP joint. Surgical repair is proposed by some authors if an injury of the collateral ligaments, capsule, and/or volar plate are present, in order to prevent later instability of the joint [6, 8, 9].

As recommended by the majority of the authors, the appropriate treatment after a successful reduction without accompanied avulsion fractures and joint instability is to immobilize the finger in an intrinsic plus position with a splint [2, 3, 10, 12] or to immobilize the injured finger with the correct adjacent finger for support (e.g., little finger with ring finger, middle finger with index finger) and mobilization as tolerated [1, 7, 11, 13]. Most authors agree that a period of immobilization between 2 and 3 weeks is sufficient and beneficial, in order to protect the healing process and parallel prevent joint stiffness [1, 2, 10, 12]. As a result, early mobilization is recommended, since it helps to minimize immobility-associated complications, and it is also important for a good functional outcome.

## 4. Conclusion

Double interphalangeal joint dislocation in a single finger is rare. In uncomplicated cases, closed reduction is the treatment of choice, and simple longitudinal traction is mostly sufficient. A buddy taping followed by early mobilization usually leads to excellent functional outcomes.

## Figures and Tables

**Table 1:** List of publications of dorsal dislocation of the proximal and distal interphalangeal joints of the same finger.

Study	M/F, years	Cause of injury	Mechanism	Finger (d/n.d)	Associated injury	F/U	Outcome	
							Extension	Flexion
Present study, 2021	M, 13	Basketball	Axial compression, hyperextension	Little (n.d)	No	3 months	Full	Full
	M, 16	Football	Hyperextension	Index (d)	No	5 months	Full	Full

Clesham et al., 2020	M, 23	Hurling	Axial compression, hyperextension	Little (d)	No	3 weeks	Full	45° (D), 90° (P)
Gorelick et al., 2018	M, 41	Personal training	Hyperextension	Little (d)	AFMP	n/r	n/r	n/r
Sbai et al., 2017	M, 32	Bike	Hyperextension	Middle (d)	No	6 months	Full	Full
Abdelaal et al., 2016	M, 39	Fall	n/r	Little (n/r)	No	4 months	Full	Full

Seki et al., 2014	M, 64	Fall from height	Hyperextension	Little (n/r)	AFMP	n/r	10° lag (P)	90° (P)
	M, 66	Fall from height	Hyperextension	Little (n/r)	No	n/r	Full	Full

Uysal et al., 2014	M, 12	Football	Hyperextension	Ring (n/r)	No	6 weeks	Full	75° (D), 90° (P)
David-West, 2013	F, 28	Fall	Hyperextension	Little (d)	No	6 months	15° lag (D)	Full
Nusem and Loch, 2012	M, 14	Rugby	n/r	Little (n/r)	No	6 weeks	Full	Full

Kim et al., 2009	M, 23	Football	n/r	Little (n/r)	No	16 months	Full	Full
	M, 59	Working	n/r	Little (n/r)	AFMP	12 months	10° lag (P)	50° (D), 80° (P)
	M, 43	Fall from height	n/r	Little (n/r)	No	6 months	10° lag (P)	90° (P)
	M, 55	Working	n/r	Little (n/r)	No	15 months	Full	Full
	M, 21	Basketball	n/r	Little (n/r)	AFMP	14 months	10° lag (P)	90° (P)

Kalakoti, 2007	M, 45	Fall	Hyperextension	Little (d)	No	18 weeks	10° lag (P)	Full
Van Ransbeeck and De Smet, 2004	M, 29	Football	Hyperextension	Ring (n/r)	No	6 months	20° lag (P)	45° (D), 85° (P)
Mesmar, 2000	M, 23	Basketball	Hyperextension	Little (n/r)	No	3 weeks	Full	Full

Takami et al., 2000	M, 35	Softball	n/r	Ring (n/r)	No	3 months	Full	Full
	M, 53	Softball	n/r	Index (n/r)	AFMP	6 months	Full	55° (D), 95° (P)
	M, 26	Basketball	n/r	Little (n/r)	No	4 months	Full	Full

M: male; F: female; d: dominant; n.d: nondominant, F/U: follow-up; AFMP: avulsion fracture of middle phalanx; D: distal; P: proximal.
